# Supplementary material for: Rapid, high throughput, automated detection of SARS-CoV-2 neutralizing antibodies against native-like vaccine and delta variant spike trimers
Source: Res Sq. 2022 Feb 16:rs.3.rs-1322411. Preprint. [Version 1] doi: 10.21203/rs.3.rs-1322411/v1 (PMC8863158; doi:10.21203/rs.3.rs-1322411/v1)
Supplement: 1 [file NIHPPrs1322411v1-supplement-1.pdf]

Supplementary Figure 1.

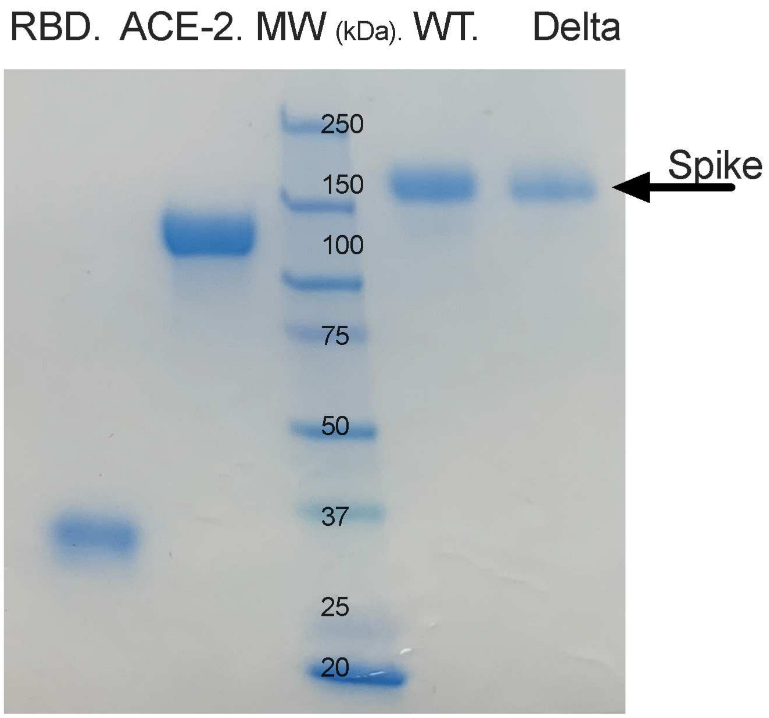

Supplementary Figure 1. SDS-PAGE for proteins from SARS-CoV-2 RBD, WT, Delta and hACE-2 expressed in Expi293 cells and purified by affinity and size exclusion chromatography.
